# LV wall motion assessment during regadenoson vasodilator stress CMR

**DOI:** 10.1186/1532-429X-18-S1-P81

**Published:** 2016-01-27

**Authors:** Michael Jesinger, Vincent L Sorrell, Steve W Leung

**Affiliations:** University of Kentucky, Lexington, KY USA

## Background

Regadenoson (REG) is a commonly used vasodilator stress agent that causes an increase in sympathetic response with associated increase in myocardial contractility and ventricular function in addition to coronary vasodilation. This agent has demonstrated high accuracy to detect coronary stenosis during myocardial perfusion imaging, but global and regional LV functional changes immediately after a REG bolus have been less well investigated. This study was designed to report the LV functional changes during routine stress perfusion CMR.

## Methods

This is a retrospective study reviewing REG stress CMR imaging obtained from 99 patients between June 2012 and February 2014. Images were obtained on a 1.5T Siemens MRI scanner (Erlangen, Germany). Pre-REG 2- and 4-chamber view steady state free precession cines were obtained. After REG perfusion stress images, repeat 2 and 4-chamber cines were obtained within 2 minutes. All studies' pre and post cine images were anonymized and randomized, and biplane end-diastolic volume and end-systolic volume were blindly assessed and LVEF calculated with CMR42 software. A change in LVEF >5% with stress was considered significant. Patients in this group were compared to patients with a non-significant change in LVEF (delta EF <=5%). These findings were correlated with the finalized CMR reports.

Medical records including cath lab and surgical reports were reviewed. Exclusion criteria: Patients with atrial fibrillation and patients without follow-up were excluded. For statistical analysis a Fisher's exact test was utilized.

## Results

Fourteen patients were excluded (5 had atrial fibrillation, and 9 had no follow-up). There were 85 patients that underwent analysis (mean age: 56 +/-12 year-old, male: 60%). Significant change in pre- and post-regadenoson stress LVEF was noted in 44 patients (Pre: 56%+/-11% vs post: 66%+/-12%) while no significant change was observed in 41 patients (Pre: 58%+/-15% vs post: 60%+/-17%).

Patients with significant increase in post-regadenoson LVEF had a trend towards lower rate of reversible perfusion defects compared to patients who did not have significant increase in EF (24.4% vs. 9.1%, p = 0.08). Patients with significant increase in post-regadenoson LVEF also had slightly lower rate of revascularization compared to patients who did not have significant increase in LVEF; however, this was not statistically significant (12.2% vs. 4.5%, p = 0.25).

## Conclusions

The change in global LVEF in stress CMR before and after regadenoson may potentially add prognostic data and help improve the sensitivity and specificity of the CMR stress test results, but not in our initial small investigation. Regional wall motion analysis and longer term follow may reveal incremental importance of wall motion assessment and are on-going.

